# Impact of socio-demographic and clinical characteristics on functional disability and health-related quality of life in patients with rheumatoid arthritis: a cross-sectional study from Palestine

**DOI:** 10.1186/s12955-021-01874-x

**Published:** 2021-10-13

**Authors:** Samah W. Al-Jabi, Diaa I. Seleit, Adnan Badran, Amer Koni, Sa’ed H. Zyoud

**Affiliations:** 1 Department of Clinical and Community Pharmacy, College of Medicine and Health Sciences, An-Najah National University, Nablus, 44839 Palestine; 2 Department of Rheumatology, Jenin Government Hospital, Palestinian Ministry of Health, Jenin, Palestine; 3 Division of Clinical Pharmacy, Hematology and Oncology Department, An-Najah National University Hospital, Nablus, 44839 Palestine; 4 Poison Control and Drug Information Center (PCDIC), College of Medicine and Health Sciences, An-Najah National University, Nablus, 44839 Palestine; 5 Clinical Research Centre, An-Najah National University Hospital, Nablus, 44839 Palestine

**Keywords:** Functional disability, HRQoL, Quality of life, Rheumatoid arthritis, Palestine

## Abstract

**Background:**

Rheumatoid arthritis (RA) is a chronic autoimmune disorder, which has a significant impact on patients' health-related quality of life (HRQoL), and limits physical function as well as increases pain and fatigue. Therefore, this study aimed to evaluate the HRQoL and functional disability profile of patients with RA in Palestine to determine the socio-demographic and clinical features associated with low HRQoL and functional disability in patients with RA and to investigate the impact of drugs used on functional disability and HRQoL.

**Methodology:**

A cross-sectional, observational study conducted at rheumatology clinics in Northern West-Bank, Palestine (Alwatani Hospital—Nablus, Khalil Suleiman Hospital—Jenin, Thabet Thatbet Hospital-Tulkarem, and Darweesh Nazzal Hospital—Qalqilia). EuroQoL-5 Dimension scale (EQ-5D-5L) was used to evaluate HRQoL, Health Assessment Questionnaire, Disability Index (HAQ-DI) to evaluate the functional disability, and the Health Assessment Questionnaire pain visual analog scale (HAQ-VAS) to evaluate pain.

**Results:**

300 patients were included in the study, 229(76.3%) were females, the mean ± standard deviation age was 49 ± 13.10 years, and the median RA duration (lower–upper quartiles) was 6 (4–12) years. The median EQ-5D-5L index value and Euro QOL visual analogue scale (EQ-VAS) scores were 0.56 and 60, respectively. There was a significant strong positive correlation (R = 0.773; *p* < 0.001) between the EQ-5D-5L index values and the reported EQ-VAS scores. The median HAQ-DI and HAQ-VAS were 0.94 and 40, respectively. The results of multiple linear regression showed that treatment with biological DMARD (Etanercept), having work, higher income, absence of night pain, and absence of comorbid diseases were significantly associated with higher EQ-5D-5L index score (better HRQoL) and lower HAQ-DI scores (less disability). On the other hand, older age and the presence of morning stiffness were significantly associated with higher HAQ-DI scores (more disability).

**Conclusions:**

This study revealed the impact of treatment, clinical variables, and socio-demographic factors on disability and HRQoL in RA patients. Healthcare providers should be aware of the association between treatment with biological DMARD and improved HRQoL and functional status to make early interventions that reduce disability and improve HRQoL in susceptible patients.

**Supplementary Information:**

The online version contains supplementary material available at 10.1186/s12955-021-01874-x.

## Background

Rheumatoid arthritis (RA) is a chronic, autoimmune, multisystemic, inflammatory, progressive condition of uncertain etiology characterized by joint pain, swelling, stiffness, and synovial joint destruction, resulting in serious impairment and premature mortality [1, 2]. It is estimated that it affects between 0.5% and 1.0% of the world's adult population and affects women more than men [3].

Rheumatoid arthritis is a troublesome condition that greatly impacts patients' lives; affecting HRQoL and limiting physical function. Fatigue is considered one of the deliberating outcomes among all chronic diseases. This impacts patients' involvement in paid jobs, their work performance within and outside the home, and their participation in family, social, and leisure activities [4].

In RA treatment, assessing health-related quality of life (HRQoL) and functional status has become an important complement to clinical, laboratory, and functional indicators in evaluating patients [5–7]. Moreover, maximizing HRQoL and maintaining physical function are treatment goals, besides early detection, intervention, controlling disease activity, and alleviating pain [6, 8].

Several tools have been proposed to evaluate the HRQoL and disability in patients with RA, including both generic tools, such as the Medical Outcomes Study 36-Item Short-Form Health Survey (SF-36), and European Quality of Life-5 Dimensions (EQ-5D); and specific tools, such as Rheumatoid Arthritis Quality of Life Scale (RAQoL), and the Health Assessment Questionnaire (HAQ) [9, 10]. Improvements in RA patients are identified by both disease-specific and standardized tests of function and HRQoL. The use of both forms of therapy evaluation measures can help recognize subtle changes that are important to patients and promote similarities between various disease states [11].

Rheumatoid arthritis disease is not being cured using the available treatment; the treatment administered can manage and control at a minimum level the disease effect on RA patients by increasing HRQoL [12]. In addition, it has been indicated that HRQoL can be considered as one of the most important components of the quality of medical and health care [13]. Few studies assessed HRQoL in Palestinian patients with other medical conditions rather than RA [14–21], and no study has assessed patients' disability in a specific disease. Despite the importance of HRQoL and the degree of disability in patients with RA, no previous studies have been carried out to the best of our knowledge to determine HRQoL or disability in patients with RA in Palestine. However, some studies have been carried out worldwide, and few regional studies have been carried out [22–33].

Therefore, this study has been conducted to conclude and provide a general and clear view of RA patients' HRQoL and disability in Palestine. In addition, the results of this study can be used in planning and interventions for improving RA patients’ HRQoL and reducing their disability. It also aims to determine the socio-demographic characteristics and clinical factors associated with poor HRQoL and functional disability of RA patients and investigate the effect of medications used on functional disability and HRQoL.

## Methods

### Study design and settings

This study is an observational cross-sectional study that included all rheumatology clinics in the government hospitals of the Northern West-Bank, Palestine. The clinics included in the study were at Alwatani Hospital—Nablus, Khalil, Suleiman Hospital—Jenin, Thabet, Thatbet Hospital—Tulkarem, and Darweesh Nazzal Hospital—Qalqilia. These clinics are working just 1 day per week by the same rheumatologist on consecutive days. They are the main care providers for RA patients in Palestine's geographical area, with approximately more than one million residents. The clinics where the study was carried out offer medical care, including rheumatologist examination, laboratory tests, radiologic imaging, and prescribing medications to be dispensed later in primary health care clinics.

### Sample size

According to data from the rheumatology clinics and the Palestinian Health Information Center, approximately 1042 RA patients were referred to the rheumatology clinics in Northern West-Bank, Palestine in the year 2012. In addition, Alwatani Hospital—Nablus received approximately 31.2% of the total visits, followed by Khalil Suleiman Hospital—Jenin (30.8%), Thabet Thatbet Hospital-Tulkaram (24%), and Darweesh Nazzal Hospital—Qalqilia (14%). Thus, according to these numbers, the sample size was calculated depending on the sample size calculator by Raosoft. The minimum effective sample size calculated for this investigation was 281 RA patients, based on a response distribution of 50% for patients with low HRQoL or functional impairment and a 5% margin of error at the 95% confidence interval. In addition, to decrease false results and maximize the reliability of the current study, the estimated sample size was increased by 5–10%.

### Data collection

Data was collected by a clinical pharmacist who is a primary health care team member and is familiar with the work system at clinics. All patients that presented at the clinic on the day that the clinical pharmacist was there approached to participate in the study were reviewed and chosen conveniently. Data was collected 4 days per week, from March to June 2014. The researcher who was in charge reached the clinic, remained in the clinic throughout the working time, interviewed each patient for at least 15 min, and assessed patients’ profiles. To assess the reliability and validity of the data collection form, a pilot test with 20 patients was carried out. Patients who participated in the pilot study were excluded from the target study sample.

### Tools used in data collection

A data collection form consists of four parts. The first part contains the socio-demographic characteristics, including age, gender, height, weight, residency, education level, marital status, work and employment status, family monthly income, and cigarette smoking.

The second part contains disease-related characteristics including age at onset, length of RA, duration (years), presence of delayed diagnosis (defined as a delay in time between the onset of symptoms and the beginning of treatment (months)), encountering of night pain (number of awakenings), morning stiffness (minutes), comorbid diseases, and medication used (corticosteroids, DMARDs, and biological agents).

The third part contains the European Quality of Life 5-Dimensions scale (EQ-5D-5L). We used EQ-5D-5L version, which were created in 2009 to improve the tool’s sensitivity, compared to the 3L version [34]. The EQ-5D-5L consists of a 5-item descriptive system to measure the EQ-5D-5L index and the EQ visual analogue scale (EQ-VAS) to estimate the patients' current health status from zero to one hundred [34]. The Arabic version of EQ-5D-5L seems to be reliable and valid in estimating HRQoL [35]. Moreover, the Cronbach's alpha value of the EQ-5D-5L instrument in the current study was found to be 0.88; that showed the high reliability of the EQ-5D-5L instrument.

The fourth part of the data collection form contains the HAQ-DI, which was used to evaluate the functional disability. HAQ-DI is the main frequent scale used for assessing functional status and disability in RA patients [36–39]. The Arabic HAQ is a reliable and valid instrument that can be self-administered to Arabic RA patients to evaluate their functional disability [40]. Moreover, the Cronbach's alpha value of the HAQ-DI tool in the current study was found to be 0.90; that showed the high reliability of the HAQ-DI tool. The scale includes 20 items that describe the difficulty that encounters respondents in their usual daily activities. In addition, the HAQ-DI score ranges from 0 (no disability) to 3 (greatest disability) and sets patients into one of three categories (mild, moderate, and severe disability) [36–38, 41].

The final section consists of the HAQ-VAS Pain Scale. This scale obtained information on how RA patients felt pain over the past week. It horizontal VAS with endpoints labeled from zero (no pain) to one hundred (worst pain) [41].

### Inclusion and exclusion criteria

The Inclusion criteria for this were as follows: (1) patients with confirmed RA diagnosis (according to the American College of Rheumatology Criteria) for at least six months and visiting rheumatology clinics in Northern West-Bank, Palestine; (2) patients who were 18 years old and above; and (3) Patients who agreed to participate. This study's exclusion criteria were as follows: (1) patients who have necrotic vascular disease, or they were handicapped prior to RA [42]; and patients who were not treated.

### Ethical approval

Before the start of the research, the Institutional Review Board (IRB)’s approval, the permission of local health authorities, and the Faculty of Graduate Studies' agreement at An-Najah National University were received to ascertain patients' rights and facilitate the research progression. Only patients who agreed to participate were included in the study after discussing the research objectives and protocols and obtaining a verbal agreement.

### Statistical data analysis

All data were analyzed using the Statistical Package for Social Sciences (SPSS) (SPSS Inc., Chicago, IL, USA) program version 16. The descriptive analysis presented normally distributed continuous variables as means ± standard deviation (SD), not normally distributed continuous variables as medians (lower–upper quartiles), and categorical variables as frequencies and percentages. Kolmogorov–Smirnov test was used to assess data normality. Differences in score results were evaluated using the t-test for continuous variables (normally distributed). The Mann–Whitney U test or Kruskal–Wallis has performed appropriately for not normally distributed ones. Either the Chi-square or the Fisher exact test was used, as appropriate, to test the significance between categorical variables. The correlation was assessed using Spearman's correlation coefficient. Multiple linear regression was used to estimate which variables were significantly related to HRQoL. Dummy variables were created for categorical variables to include them in the multiple linear regression. On the other hand, the reliability of the study scales was assessed using Cronbach's alpha values. The level of significance was determined at *p* < 0.05. In addition, the scores of EQ-5D-5L were calculated depending on values taken from the United Kingdom general population using the EQ-5D-5L Crosswalk Index Calculator (http://www.euroqol.org/about-eq-5d/valuation-of-eq-5d/eq-5d-5l-value-sets.html accessed August 2014). United Kingdom tariff for the EQ-5D-5L was used to derive the EQ-5D-5L index values as Palestinian tariff was not available. It was also used in previous publications conducted in Palestine [14, 17, 21, 43, 44].

## Results

### Socio-demographic characteristics of the study patients

A total number of 300 patients with RA were included in this study. Table 1 shows the socio-demographic characteristics of the patients. The mean age (± SD) of the patients was 49 ± 13.1 years, ranging from 18 to 89. The majority of patients were females (76.3%), giving a female: male ratio of 3.2:1. In addition, 183 (61%) patients were living in villages, and 91 (30.3%) were living in urban areas.

**Table 1:** Socio-demographic characteristics of the study patients (N = 300)

Variable	Mean ± SD (range) Or N (%) Total = 300
Age (year)	49 ± 13.10 (18–89)
*Rheumatology clinic*	
Nablus	94 (31.3%)
Jenin	93 (31%)
Tulkarm	72 (24%)
Qalqilia	41 (13.7%)
*Gender*	
Male	71 (23.7)
Female	229 (76.3)
*Residency*	
City	91 (30.3)
Village	183 (61)
Refugee camp	26 (8.7)
*Conjugal status*	
With a spouse/partner	233 (77.7)
Without a spouse/partner	67 (22.3)
Single	46 (15.3)
Divorced	5 (1.7)
Widowed	16 (5.3)
*Level of education*	
Illiterate	33 (11)
Primary	128 (42.7)
Secondary	81 (27)
University	58 (19.3)
*Employment status*	
Working	79 (26.3)
Employee	41 (13.7)
Private job	38 (12.7)
Not working	221 (73.7)
Unemployed	18 (6)
Housewife	203 (67.7)
*Income (NIS)*	
Low (Less than 2000)	133 (44.3)
Moderate (2000–5000)	167 (55.7)
High (More than 5000)	2 (0.7)
*BMI*	
Normal (18.5–24.9)	104 (34.7)
Overweight (25–29.9)	105 (35)
Obese (30 or greater)	91 (30.3)
*Smoking*	
Current smoking	22 (7.3)
Never smoke	258 (86)
Ex-smoker	20 (6.7)

*BMI* body mass index, *NIS* new Israeli shekel, *SD* standard deviation

### Clinical characteristics of RA patients

Table 2 shows the RA related clinical characteristics of patients. The mean (± SD) age of RA onset was 40.61 ± 13.44 years, with a range from 10 to 77 years. Among the patients included, 192 (64%) patients had a delayed diagnosis; 186 patients of them had a median (interquartile range) duration of delay of 12 (4–12) months. Regarding their daily symptoms, the majority of patients (65.3%) had morning stiffness. In addition, 196 (65.3%) patients had night pain affecting their sleep, causing awakenings each night. Regarding the presence of other comorbid diseases, 172 (57.3%) patients had comorbid conditions.

**Table 2:** Clinical characteristics of the study patients

Variable	Mean ± SD Median (lower–upper quartile) Or N (%) Total = 300
Age of RA onset (years)	40.61 ± 13.44 (10–77)
RA duration (years)	6 (4–12)
*Diagnosis delay*	
Delay	192 (64)
No delay	108 (36)
Delay diagnosis (months)^a^	12 (4–12)
*Morning stiffness*	
Stiffness	196 (65.3)
No stiffness	104 (34.7)
Morning stiffness (minutes)	15 (10–30)
*Night pain*	
Pain	196 (65.3)
No pain	104 (34.7)
Number of awakenings	2 (2–3)
*Comorbid diseases*	
Yes	172 (57.3)
No	128 (42.7)
Number of comorbid diseases	2 (1–2)
*Number of comorbid diseases*	
1	75 (25)
2	55 (18.3)
3	24 (8)
4	12 (4)
5	6 (2)

*RA* rheumatoid arthritis

^a^Data for delayed diagnosis were only available for 186 patients

### Medications used among RA patients

Methotrexate was the most commonly used conventional DMARD; it was used by 224 (74.4%) patients, followed by Leflunomide (17%), hydroxychloroquine (11.3%), sulfasalazine (5.3%), and azathioprine (4%). In addition, regarding biotherapy, 37 (12.3%) patients received etanercept, either alone or in combination with conventional DMARDs. In addition, prednisolone was used in most therapeutic plans; 260 (86.7%) patients received it (Table 3).

**Table 3:** medications used to treat RA patients

Variable	Mean ± SD (range), median (lower–upper quartile) Or N (%) Total = 300
*Medications*	
Corticosteroids (Prednisolone)	260 (86.7)
DMARDs	279 (93)
Methotrexate	224 (74.4)
Leflunomide	51 (17)
Hydroxychloroquine	34 (11.3)
Sulfasalazine	16 (5.3)
Azathioprine	12 (4)
Biotherapies (Etanercept)	37 (12.3)
*Therapeutic plan*	
Single DMARD	202 (67.3)
Combination of 2 DMARDs	61 (20.3)
Biotherapy (Etanercept)	22 (7.3)
Combination of Etanercept and DMARD	15 (5)

### EQ-5D-5L index score

The median of the EQ-5D-5L index was 0.56 (IQR: 0.33–0.74). Patients aged more than 60 years significantly had the lowest EQ-5D-5L index score (*p* < 0.001). Male patients had a significant higher median EQ-5D-5L index score than females (*p* < 0.001). Patients with a higher level of education, employed patients, and those with moderate or high income had significantly higher median EQ-5D-5L index scores. Regarding residency; patients living in refugee camps had significant higher median EQ-5D-5L index scores than those living in cities or patients living in villages (*p* = 0.018) (Additional file 1: Table S1).

Furthermore, patients who complained of morning stiffness, night pain, comorbid diseases, or had a delay in diagnosis had significantly lower median values of EQ-5D-5L index score (Additional file 1: Table S2).

On the other hand, patients treated with the biological agent (Etanercept) had significant higher median EQ-5D-5L values (*p* < 0.001). However, patients treated with conventional DMARDs had significantly lower median values of EQ-5D-5L (*p* < 0.001). Regarding the therapeutic plan, the patients followed it; the results showed that patients using a biological agent (Etanercept) significantly had the highest EQ-5D-5L median score (0.82 (0.73–1)) (Additional file 1: Table S3).

On the other hand, the results show a significantly weak negative association between EQ-5D-5L index values and RA duration (R = − 0.167; *p* = 0.006), In addition to, age of RA onset (R = − 0.158; *p* = 0.006).

### EQ-visual analogue (VAS)

The median of EQ-VAS was 60 (IQR: 50–77.5). Older patients significantly had the lowest EQ-5D-5L index score (*p* < 0.001). Significant differences were also shown in variables such as gender (*p* < 0.001) and BMI (*p* < 0.001). Furthermore, treatment with the biological agent (Etanercept) was significantly associated with higher median values of VAS (*p* < 0.001).

### *Correlation between the* EQ-5D-5L *index and EQ-VAS*

The EQ-5D-5L index was significantly and positively correlated (R = 0.773; *p* < 0.001) with the reported EQ-VAS scores.

### Multiple linear regression analysis for HRQoL

Further analysis was done using multiple linear regression to examine which variables were significantly related to HRQoL (R = 0.654; adjusted R2 = 0.418; F = 15.7; df = 13; *p* < 0.001). Table 4 shows that treatment with biological DMARD (Etanercept), work, higher income, and absence of night pain, absence of comorbid diseases were significantly associated with higher EQ-5D-5L index score (better HRQoL).

**Table 4:** Association between factors and EQ-5D-5L score using multiple linear regression analysis

Variables	Standardized coefficients (beta)	*p *value
*Age*		
Continuous (1-year units)	− 0.082	0.124
*Gender*		
Male	0.083	0.174
Female	Ref	
*Body mass index*		
Normal	− 0.053	0.309
Overweight or obese	Ref	
*Residency*		
City	0.084	0.076
Others	Ref	
*Work status*		
Unemployed	− 0.135	0.036
Employed	Ref	
*Income*		
Moderate and High	0.148	0.004
Low	Ref	
*Education*		
University	0.034	0.586
No formal, primary, secondary	Ref	
*Diagnosis delay*		
No	0.072	0.132
Yes	Ref	
*Morning stiffness*		
No	0.076	0.185
Yes	Ref	
*Night pain*		
No	0.276	< 0.001
Yes	Ref	
*Comorbid diseases*		
No	0.211	< 0.001
Yes	Ref	
*Conventional DMARDs*		
No	− 0.033	0.603
Yes	Ref	
*Etanercept use*		
No	− 0.153	0.017
Yes	Ref	

*DMARDs* disease-modifying antirheumatic drugs, *EQ-5D* European Quality of life scale, *SE* standard error

### Disability of RA patients

According to HAQ-DI scores, the median (interquartile range) of HAQ-DI scores was 0.94 (0.5–1.5). According to HAQ-DI categories, patients were categorized into three categories (mild to moderate, moderate to severe, and severe to very severe disability). Our data shows that half of the patients (50.0%) had mild to moderate disability. Disability was significantly associated with morning stiffness (*p* < 0.001), night pain (*p* < 0.001), and comorbid diseases (*p* < 0.001) (Additional file 1: Table S4).

Of note, patients treated with the biological agent (Etanercept) were having less disability significantly (*p* < 0.001). However, treatment with conventional DMARDs had significant severe to very severe disability (*p* = 0.003). Concerning the treatment strategy, patients using a combination of a biological agent (Etanercept) and conventional DMARDs or those who were using a biological agent (Etanercept) alone significantly complained of mild to moderate disability (*p* < 0.001) (Additional file 1: Table S5). Moreover, the results show a significantly weak positive association between HAQ-DI and RA duration (R = 0.191; *p* = 0.001). In addition, a significantly moderate positive association was found between HAQ-DI and age of RA onset (R = 0.324; *p* < 0.001).

### Multiple linear regression analysis for disability

To assess which variables were significantly related to HAQ-DI scores, multiple linear regression analysis was applied (R = 0.666; adjusted R2 = 0.418; F = 17.5; df = 13; *p* < 0.001). Significantly associated with higher HAQ-DI scores (disabled) were found in the following factors: older patients, being without work, lower income, presence of morning stiffness, presence of night pain, presence of comorbid diseases, and patients not treated with biological DMARD (Etanercept) (Table 5).

**Table 5:** Association between factors and HAQ-DI score using multiple linear regression analysis

Variables	Standardized coefficients (beta)	*p *value
*Age*		
Continuous (1-year units)	0.226	< 0.001
*Gender*		
Male	− 0.047	0.429
Female	Ref	
*Body mass index*		
Normal	0.029	0.566
Overweight or obese	Ref	
*Residency*		
City	− 0.056	0.227
Others	Ref	
*Work status*		
Unemployed	0.127	0.042
Employed	Ref	
*Income*		
Moderate and high	− 0.130	0.010
Low	Ref	
*Education*		
University	0.001	0.990
No formal, primary, secondary	Ref	
*Smoking*		
No	− 0.078	0.084
Yes	Ref	
*Morning stiffness*		
No	− 0.157	0.005
Yes	Ref	
*Night pain*		
No	− 0.148	0.008
Yes	Ref	
*Comorbid diseases*		
No	− 0.218	< 0.001
Yes	Ref	
*Conventional DMARDs*		
No	− 0.009	0.888
Yes	Ref	
*Etanercept use*		
No	0.164	0.009
Yes	Ref	

*DMARDs* disease-modifying antirheumatic drugs, *HAQ-DI* health assessment questionnaire disability index, *SE* standard error

### HAQ-VAS pain scale

The median of HAQ-VAS pain was 40 (IQR: 20–50). There was a significantly strong negative correlation (R = − 0.622; *p* < 0.001) between the HAQ-VAS pain scores and the reported EQ-VAS scores. Furthermore, a significantly strong positive association was observed between HAQ-DI and HAQ-VAS for pain (R = 0.661; *p* < 0.001).

### Relationship between HRQoL and disability scores

This study revealed a significant strong negative association between HRQoL and disability (HAQ-DI), according to EQ-VAS (R = − 0.800; *p* < 0.001) and EQ-index (R = − 0.775; *p* < 0.001).

## Discussion

The present study introduced extensive information about HRQoL and functional disability among RA patients in the Northern West Bank, Palestine. HRQoL and disability were estimated using the EQ-5D-5L and HAQ-DI, respectively. Referring to the literature, EQ-5D-5L has been used to evaluate HRQoL in RA patients, and it is valid, reliable, and responsive in both specific and general disease populations. HAQ-DI is the most frequently used scale of disability in RA patients.

The mean age of our sample was 49 years, 77% were married, and 7.3% were smokers. The majority of patients were females (76.3%), giving a female: male ratio of 3.2:1, with a median disease duration of 6 years. Palestinians belong to Arabic nations, and the state of Palestine locates in the Middle East and North Africa region. The reported prevalence of RA in this region was 0.16%, with an estimation of RA distribution among genders being 3:1 [45], which is similar to our sample. The characteristics of the RA in the United Arab Emirates previous study were as follows: 4: 1 female to male ratio, 11% were smokers, and 89% were married [46]. In the Saudi Arabia study, 85.6% of the patients were female, 40.49 years was the mean age, with a mean disease duration of 5.51 years [47].

Notably, age, gender, pain, income, education level, employment status, social status, and body mass index were all previously noted to impact the quality of life of rheumatoid arthritis patients [27, 48–50]. In addition, other factors such as disease activity, depression, tiredness, anxiety, sleep duration, psychological counseling, and C4 and IgA levels also influence the QoL of RA patients [27, 48].

In the current study, numerous patients’ characteristics were found to be related to poor HRQoL in RA patients, such as not working, low income, pain, comorbid diseases, and nonbiological DMARDs use. Furthermore, although residents of refugee camps have higher EQ-5D-5L index values than urban or rural residents, confounding characteristics might affect the EQ-5D-5L index. Additionally, a small number of patients (n = 26; 8.7%) who reside in refugee camps were included in our sample.

In this study, the median of EQ-5D-5L scores among RA patients was 0.56, while results from previous studies that used the EQ-5D-5L in Danish, Hungarian, Korean, Japanese, and Swedish patients were 0.72 [51], 0.46 [52], 0.60 [53], 0.76[54], and 0.60[55], respectively. The median of EQ-VAS scores was 60. Moreover, the scores of EQ-VAS were strongly and positively correlated to EQ-5D-5L index values. Using different measures such as the EQ-5D-5L and EQ-VAS to assess individual experience may result in slightly different outcomes, and accuracy in outcomes when using EQ-5D-5L is probable than EQ-VAS[14].

On the other hand, the current data showed that being unemployed or with low income was significantly associated with lower HRQoL. These results were emphasized in previous studies and considered a significant variable that may deteriorate HRQoL in RA patients [48, 56–59]. Gamal et al. [59] mentioned that working is an advantage for individuals as it makes them able to earn income and gives a sense of accomplishing activities that contribute to society. Importantly, this factor seems to be confounder as poor quality of life might be why patients with RA leave the labor force.

The present results revealed that a higher pain degree was related to lower HRQoL. This result was supported by previous studies [23, 48, 53]. The findings demonstrated that night pain, evaluated by the number of sleep awakenings, was related to lower HRQoL. Purabdollah et al. [60] and Sariyildiz et al. [61] found that sleep problems negatively affected HRQoL in RA patients. A possible explanation is that sleep disturbances are affected by age, disease activity, functional disability, duration of morning stiffness, and the presence of these factors lower HRQoL in RA patients [61]. As for clinical factors, HRQoL was worsened by the existence of comorbidities. A similar relation was confirmed in previous studies [49, 56, 57]. Moreover, comorbid conditions may affect the selection of therapy regimen, or the used medications may deteriorate the comorbidity [62].

The current findings revealed that patients managed with the biological DMARD, etanercept, had higher HRQoL than patients treated with conventional DMARDs. Similar results were observed in other studies [2, 57, 63]. Moreover, patients who were refractory to conventional DMARDs had improvements in HRQoL with the use of biological DMARDs [64, 65]. Furthermore, regarding the combination of etanercept with methotrexate, the results showed that the combination of etanercept with methotrexate was having higher HRQoL than other therapeutic strategies. However, in a previous study, combining etanercept with methotrexate in the treatment regimen was mostly superior to either monotherapy in improving HRQoL [66].

In the current research, elderly, unemployment, low-income, morning stiffness, night pain, comorbid diseases, nonbiological DMARDs use were all predictors of poor functional disability.

Regarding HAQ-DI scores, the average score that has been reported in RA patients is 1.2 [41]. In the current study, it has been found that the median HAQ-DI score among RA patients was 0.94, while the scores from studies that used HAQ-DI in Egyptian patients were 1.08[67] and 1.45 [68]. In Moroccan patients, the median HAQ-DI scores among RA patients were 1.40[69], and 1.63 [23], while in Tunisian patients, the score was 1.7 [70].

In the current study, it has been found that older RA patients had worse disability than younger ones, which was relevant to previous studies [71–76]. A possible explanation for this finding was that older RA patients had a longer duration of the disease and more comorbid conditions [77], and these characteristics worsen the functional status.

In the current study, the presence of morning stiffness was associated with lower functional status. This result was observed in previous researches [78, 79]. Night pain, evaluated by the number of sleep awakenings, was associated with functional disability [61, 80]. Sleep problems in RA sufferers may result from pain, depression, and more active RA [81], and these factors participate in the worsening of disability in RA patients.

As for clinical factors, the presence of comorbidity was found to be related to disability. A similar finding was observed in previous studies [76, 82, 83]. Comorbid conditions are more common in the elderly population. Moreover, patients suffering from multiple comorbidities were treated less aggressively, or prescribed drugs may worsen their comorbidities [62].

On the other hand, the present study indicated that patients treated with the biological DMARD, etanercept, had less disability than patients treated with conventional DMARDs. This result was in agreement with the result in Genovese et al. [84] study. Moreover, etanercept therapy significantly improved disability in RA patients after failure of conventional DMARD therapy [85–87].

Palestinian health care providers should pay more attention to certain patients’ characteristics, such as age, income status, and comorbid diseases. In general, biological DMARDs could be offered to a larger number of RA patients in accordance with risk, benefit, and cost assessment, as QoL and disability are important outcomes in these populations. Moreover, extensive research should be conducted to evaluate the use of biological DMARDs in RA, considering other important factors affecting the intended outcomes. In Palestine, the choice of treatment regimen depended on the availability of medications and, to some extent, the overall cost, as Palestine is under occupation and not all materials were easily accessible. That, unfortunately, biological DMARDS could not be reasonable for many RA patients.

Since 2014 and until now, some changes have occurred in the status of RA in Palestine. First, the number of rheumatologists has increased, which helped improve the awareness of RA among the general public and health care practitioners that resulted in early diagnosis of the disease. Additionally, new medications have become available at the Palestinian Ministry of Health.

## Strengths and limitations

To the extent of our knowledge, this research is the first in Palestine regarding RA and its impact on HRQoL and functional disability, providing a clear view into an unstudied field and initiating a database for future studies focusing on RA patients in Palestine. The study included all RA centers in the northern West Bank of Palestine using the target sample size. Furthermore, the data were recruited via face-to-face interview, giving complete data.

However, the current study had some limitations. First, because of the cross-sectional design, we could not determine a causal relationship between treatment effects, e.g., DMARDs, and the outcomes. Second, the sample population was selected by a convenience sampling method that may affect results generalization. Third, a limitation of the face-to-face interview is the presence of bias as patients may wish to give a private response. However, face-to-face interviews have some advantages: they give more accurate screening, keep the patient-focused while answering, capture verbal and nonverbal cues, and capture behavior and emotions. In addition, the quality of the questions of this study can be answered without any embarrassment. Fourth, some limitations were associated with a poor recall of RA patients' experiences. Lastly, the study lacked some clinical variables such as laboratory tests, disease activity measures, and imaging findings. Their presence might give more data about RA in Palestine and reveal possible factors related to functional status or HRQoL in RA patients.

## Conclusions

The current study was designed to evaluate functional disability and HRQoL among RA patients, determine factors related to disability and poor HRQoL, and assess treatment regimens' influence on disability and HRQoL. The results revealed many important factors that must be considered when providing care to RA patients.

The study found that the median EQ-5D-5L index scores in RA patients were approximately half the total score. In addition, work, higher income, absence of night pain, absence of comorbid diseases, and treatment with biological DMARD (Etanercept) were significantly associated with higher EQ-5D-5L index score (better HRQoL). In addition, it has been found that the disability score (HAQ-DI score) among RA patients was high. Furthermore, older patients, being without work, lower income, presence of morning stiffness, presence of night pain, presence of comorbid diseases, and patients not treated with biological DMARD (Etanercept) were significantly associated with higher HAQ-DI scores (disabled). On the other hand, patients with poor HRQoL were more disabled. Based on our findings, we recommend: (1) recommend routine assessment of HAQ-DI or EQ-5D-5L to detect changes through the course of RA and the need for intensive treatment; (2) rheumatologists, clinical pharmacists, and other healthcare workers should be aware of the association between treatment with biological DMARD and improved HRQoL and functional status, to make early interventions that reduce disability and improve HRQoL in susceptible patients; and (3) future studies regarding RA should be encouraged to burden the knowledge in this field, to investigate the effect of unstudied factors on HRQoL and functional status among RA patients, and to cover other geographical regions in Palestine.

## Supplementary Information

**Additional file 1: Table S1**. Socio-demographic characteristics of the study patients with differences in health-related quality of life (HRQoL). **Table S2** Rheumatoid arthritis-related clinical characteristics of the study patients with differences in health-related quality of life (HRQoL). **Table S3** Rheumatoid arthritis treatment characteristics of the study patients with differences in health-related quality of life (HRQoL). **Table S4** Rheumatoid arthritis-related clinical characteristics of the study patients with differences in disability categories. **Table S5** Rheumatoid arthritis treatment characteristics of the study patients with differences in disability categories.

## Data Availability

Data and materials used in the study of this manuscript are available from the corresponding author upon request.

## Abbreviations

IRB: Institutional Review Board
ACR: The American College of Rheumatology
AIMS2-SF: Arthritis Measurement Scale 2-Short Form
ACPA: Anti-citrullinated protein, antibody
BMI: Body mass index
cDMARDs: Combination disease-modifying antirheumatic drugs
CI: Confidence interval
CRP: C-reactive protein
DAS: Disease activity scores
DMARDs: Disease-modifying antirheumatic drugs
EQ-5D: European Quality of Life-5 Dimensions
EQ-VAS: The European Quality Visual Analogue Scale
ESR: Erythrocyte sedimentation rate
EULAR: The European League Against Rheumatism
HAQ: Health Assessment Questionnaire
HAQ-DI: Health Assessment Questionnaire Disability Index
HAQ-VAS: Health Assessment Questionnaire Visual Analogue Scale
HRQoL: Health Related Quality of Life
MDHAQ: Modified Health Assessment Questionnaire
NIS: New Israeli Shekel
OA: Osteoarthritis
OR: Odds ratio
RA: Rheumatoid arthritis
RAQoL: Rheumatoid Arthritis Quality of Life Scale
RF: Rheumatoid factor
SD: Standard deviations
SF-12v2: The Short Form 12 Items (Version 2) Health Survey
SF-36: Medical Outcomes Study 36-Item Short-Form Health Survey
SPSS: Statistical Package for Social Sciences
TNFi: Tumor necrosis factor inhibitors
WHO: World Health Organization
