# Supplementary material for: Impact of socio-demographic and clinical characteristics on functional disability and health-related quality of life in patients with rheumatoid arthritis: a cross-sectional study from Palestine
Source: Health Qual Life Outcomes. 2021 Oct 13;19:241. doi: 10.1186/s12955-021-01874-x (PMC8513295; doi:10.1186/s12955-021-01874-x)
Supplement: Supplementary file 1 — Additional file 1: Table S1. Socio-demographic characteristics of the study patients with differences in health-related quality of life (HRQoL). Table S2 Rheumatoid arthritis-related clinical characteristics of the study patients with differences in health-related quality of life (HRQoL). Table S3 Rheumatoid arthritis treatment characteristics of the study patients with differences in health-related quality of life (HRQoL). Table S4 Rheumatoid arthritis-related clinical characteristics of the study patients with differences in disability categories. Table S5 Rheumatoid arthritis treatment characteristics of the study patients with differences in disability categories. [file 12955_2021_1874_MOESM1_ESM.doc]

**Additional file 1: Table S1. Socio-demographic characteristics of the study patients with differences in health-related quality of life (HRQoL). Table S2** Rheumatoid arthritis-related clinical characteristics of the study patients with differences in health-related quality of life (HRQoL). **Table S3** Rheumatoid arthritis treatment characteristics of the study patients with differences in health-related quality of life (HRQoL). **Table S4** Rheumatoid arthritis-related clinical characteristics of the study patients with differences in disability categories. **Table S5** Rheumatoid arthritis treatment characteristics of the study patients with differences in disability categories.

**Table S1 Socio-demographic characteristics of the study patients with differences in health-related quality of life (HRQoL)**

| **Variable** | **EQ5D index Score**  **Median (IQR)** | **P-value** | **EQ-VAS Median (IQR)** | **P-value** |
| --- | --- | --- | --- | --- |
| **Age**  < 30 years  30-60 years  ˃ 60 years | 0.86 (0.68-0.98)  0.59 (0.41-0.74)  0.43(0.16-0.59) | < 0.001^b^ | 85 (80-90)  70 (50-80)  50 (40-70) | < 0.001^b^ |
| **Gender**  Male  Female | 0.68 ( 0.52-0.87)  0.54 (0.32-0.69) | < 0.001^a^ | 70 (60-85)  60 (50-70) | < 0.001^a^ |
| **BMI**  Normal  Overweight  Obese | 0.69 (0.49-0.83)  0.55(0.35-0.66)  0.42(0.20-0.58) | < 0.001^b^ | 70 (60-85)  60 (50-70)  50 (50-70) | < 0.001^b^ |
| **Residency**  City  Village  Refugee camp | 0.61 (0.31-0.76)  0.55 (0.32-0.68)  0.64 (0.51-0.83) | 0.018^b^ | 70 (50-80)  60 (50-70)  70 (50-85) | 0.112^b^ |
| **Employment status**  Working (employee or private job)  Not working (unemployed or housewife) | 0.72 (0.62-0.87)  0.53 (0.31-0.64) | < 0.001^a^ | 70 (60-85)  60 (50-70) | < 0.001^a^ |
| **Conjugal status**  With a spouse/partner  Without a spouse/partner | 0.57 (0.40-0.73)  0.55 (0.22-0.67) | 0.056^a^ | 65 (50-80)  60 (45-70) | 0.018^a^ |
| **Income**  Low  Moderate and High | 0.48 (0.20-0.62)  0.64 (0.48-0.79) | < 0.001^a^ | 50 (50-70)  70(60-80) | < 0.001^a^ |
| **Level of education**  Illiterate  Primary  Secondary  University | 0.20 (0.12-0.55)  0.53 (0.32-0.63)  0.64 (0.48-0.76)  0.73 (0.64-0.87) | < 0.001^b^ | 50 (25-50)  60 (50-70)  70 (60-85)  75 (70-85) | < 0.001^b^ |
| **Smoking**  Current smoking  Never smoke  Ex-smoker | 0.46 (0.20-0.75)  0.55 (0.36-0.72)  0.68 (0.43-0.80) | 0.236^b^ | 60 (50-70)  60 (50-80)  75 (55-85) | 0.103^b^ |

^a^ Calculated by using the Mann-Whitney U test

^b^ Calculated by using the Kruskal-Wallis test

**Table S2 Rheumatoid arthritis-related clinical characteristics of the study patients with differences in health-related quality of life (HRQoL)**

| **Variable** | **EQ5D index Score**  **Median (IQR)** | **P-value** | **EQ-VAS Median (IQR)** | **P-value** |
| --- | --- | --- | --- | --- |
| **Delay in diagnosis**  Yes  No | 0.55 (0.32-0.72)  0.61 (0.40-0.76) | 0.026 ^a^ | 60 (50-70)  65 (50-80) | 0.055^a^ |
| **Morning stiffness**  Yes  No | 0.51 (0.25-0.64)  0.73 (0.59-0.84) | < 0.001^a^ | 60 (50-70)  72 (60-85) | < 0.001 ^a^ |
| **Night pain**  Yes  No | 0.49 (0.23-0.64)  0.75 (0.60-0.84) | < 0.001^a^ | 57 (50-70)  75 (70-85) | < 0.001 ^a^ |
| **Comorbid diseases**  Yes  No | 0.51 (0.20-0.64)  0.66 (0.53-0.83) | < 0.001^a^ | 55 (50-70)  70 (60-85) | < 0.001 ^a^ |

^a^ Calculated by using the Mann-Whitney U test

**Table S3 Rheumatoid arthritis treatment characteristics of the study patients with differences in health-related quality of life (HRQoL)**

| **Variable** | **EQ5D index Score**  **Median (IQR)** | **P-value** | **EQ-VAS Median (IQR)** | **P-value** |
| --- | --- | --- | --- | --- |
| **Medications**  **Corticosteroids** (Prednisolone)  Yes  No  **DMARDs**  Yes  No  **Biotherapies** (Etanercept)  Yes  No | 0.57 (0.38-0.73)  0.47 (0.28-0.64)  0.55 (0.32-0.72)  0.79 (0.73-0.87)  0.76 (0.73-1.00)  0.55 (0.32-0.67) | 0.113 ^a^  <0.001^a^  <0.001^a^ | 65 (50-80)  60 (50-70)  60 (50-75)  75 (70-85)  80 (70-90)  60 (50-70) | 0.184 ^a^  0.002 ^a^  < 0.001^a^ |
| **Therapeutic plan**  Single DMARD  Combination of 2 DMARDs  Biotherapy (Etanercept)  Combination of Etanercept and- DMARD | 0.53 (0.32-0.65)  0.63 (0.45-0.78)  0.82 (0.73-1)  0.75 (0.73-1) | <0.001 | 60 (50-70)  70 (50-80)  77.5 (68.8-85)  85 (70-90) | < 0.001^b^ |

^a^  Calculated by using the Mann-Whitney U test

^b^ Calculated by using the Kruskal-Wallis test

**Table S4** Rheumatoid arthritis-related clinical characteristics of the study patients with differences in disability categories

| **Variable** | **Disability category** | | | P-  value |
| --- | --- | --- | --- | --- |
|  | **Mild to moderate** | **Moderate to severe** | **Severe to very severe** |  |
| **Delay in diagnosis**  Yes  No | 86 (57.3)  64 (42.7) | 78 (70.9)  32 (29.1) | 28 (70)  12 (30) | 0.055 ^a^ |
| **Morning stiffness**  Yes  No | 70 (46.7)  80 (53.3) | 94 (85.5)  16 (14.5) | 32 (80.0)  8 (20.0) | < 0.001 ^b^ |
| **Night pain**  Yes  No | 71 (47.3)  79 (52.7) | 91 (82.7)  19 (17.3) | 34 (85.0)  6 (15.0) | < 0.001^b^ |
| **Comorbid diseases**  Yes  No | 61 (40.7)  89 (59.3) | 74 (67.3)  36 (32.7) | 37 (92.5)  3 (7.5) | < 0.001^b^ |

^a^ Calculated by using the Chi-square test

^b^ Calculated by using Fisher's exact test

**Table S5 Rheumatoid arthritis treatment characteristics of the study patients with differences in disability categories**

| **Variable** | **Disability category** | | | P-  value |
| --- | --- | --- | --- | --- |
|  | **Mild to moderate** | **Moderate to severe** | **Severe to very severe** |  |
| **Medications**  **Corticosteroids** (Prednisolone)  Yes  No  **DMARDs**  Yes  No  **Biotherapies** (Etanercept)  Yes  No | 132 (88.0)  18 (12.0)  132 (88.0)  18 (12.0)  32 (21.3)  118 (78.8) | 94 (85.5)  16 (14.5)  107 (97.3)  3 (2.7)  4 (3.6)  106 (96.4) | 34 (85.0)  6 (15.0)  40 (100)  0 (0.0)  1 (2.5)  39 (97.5) | 0.792^a^  0.003 ^a^  < 0.001^a^ |
| **Therapeutic plan**  Single DMARD  Combination of 2 DMARDs  Biotherapy (Etanercept)  Combination of Etanercept and- DMARD | 84 (56.0)  34 (22.7)  19(12.7)  13 (8.7) | 91 (82.7)  15 (13.6)  3 (2.7)  1 (0.9) | 27 (67.5)  12 (30.0)  0 (0.0)  1 (2.5) | < 0.001^a^ |

^a^ Calculated by using Fisher's exact test
